# Oxidative Stress and Metabolic Pathologies: From an Adipocentric Point of View

**DOI:** 10.1155/2014/908539

**Published:** 2014-07-20

**Authors:** Soazig Le Lay, Gilles Simard, Maria Carmen Martinez, Ramaroson Andriantsitohaina

**Affiliations:** ^1^INSERM UMR 1063, “Stress Oxydant et Pathologies Métaboliques”, Institut de Biologie en Santé, 4 rue Larrey, 49933 Angers, France; ^2^Department of Biochemistry, Université d'Angers, CHU Angers, 4 rue Larrey, 49933 Angers Cedex 9, France

## Abstract

Oxidative stress plays a pathological role in the development of various diseases including diabetes, atherosclerosis, or cancer. Systemic oxidative stress results from an imbalance between oxidants derivatives production and antioxidants defenses. Reactive oxygen species (ROS) are generally considered to be detrimental for health. However, evidences have been provided that they can act as second messengers in adaptative responses to stress. Obesity represents a major risk factor for deleterious associated pathologies such as type 2 diabetes, liver, and coronary heart diseases. Many evidences regarding obesity-induced oxidative stress accumulated over the past few years based on established correlations of biomarkers or end-products of free-radical-mediated oxidative stress with body mass index. The hypothesis that oxidative stress plays a significant role in the development of metabolic disorders, especially insulin-resistance state, is supported by several studies where treatments reducing ROS production reverse metabolic alterations, notably through improvement of insulin sensitivity, hyperlipidemia, or hepatic steatosis. In this review, we will develop the mechanistic links between oxidative stress generated by adipose tissue in the context of obesity and its impact on metabolic complications development. We will also attempt to discuss potential therapeutic approaches targeting obesity-associated oxidative stress in order to prevent associated-metabolic complications.

## 1. Introduction

The prevalence of obesity over the past years has been in constant progression leading the World Health Organization to consider it as an epidemic pathology. Obesity is defined as an excessive accumulation of body fat mass to the extent that individual's health will be negatively affected. Indeed, obesity is considered as a top risk factor to develop deleterious associated pathologies as type 2 diabetes, liver, and coronary heart diseases.

White adipose tissue (WAT) constitutes the main energy supply in the body, which will ensure whole-body energy homeostasis by either storing excess energy in the form of lipids (namely, triacylglycerol—TG) or mobilizing fatty acids (FA) according to metabolic needs. However, the view of WAT, long-time considered as an inert storage depot, has considerably evolved since the identification of the adipocyte-secreted hormone leptin acting at the central level to control food intake [1]. This major discovery was followed by the characterization of many molecules secreted by adipocytes, called adipokines, which has revealed the endocrine potential of this tissue and its property to communicate with others [2]. Upon nutrient overloading, WAT will expand in considerable proportions through the ability of adipocyte to mechanistically adapt to increasing in their size. Thus, WAT can account for more than 50% of total body weight in obese subjects. This adipose plasticity is also supported by the constant renewal of adipocytes based on differentiation of adipocytes precursors present in WAT, which has been estimated to be about 10% per year [3]. Adipocyte represents the functional cell type specialized in lipid storage in WAT. The latter also regroups other cell types including precursors, immune cells, and endothelial cells. The rapid expansion of WAT in response to nutrient overload is signed by a profound remodeling of fat affecting all cellular components of WAT. This remodeling especially is characterized by an increased immune cells infiltration [4, 5]. Moreover, cellular stresses concomitant to fat overloading such as metabolic dysfunctions, inflammation, hypoxia, reticulum endoplasmic stress, and hypoxia will contribute to attracting and retaining inflammatory cells within the WAT (for review, [6]). The development of a chronic low-grade inflammatory state has been shown to play a central role in the development of metabolic complications associated obesity, since it has been linked to the development of insulin resistance, endothelial and microvascular dysfunctions [7]. Similar to other inflammatory processes such as microbial infection, WAT inflammation is intrinsically linked to oxidative stress.

Systemic oxidative stress is part of the numerous biological alterations reported during chronic obesity [8]. Evidences regarding obesity-induced oxidative stress are derived from several clinical studies, which have established correlations of biomarkers, or end-products of free radicals-mediated oxidative stress (lipid peroxidation or protein carbonylation products) with body mass index (BMI) [9, 10]. In contrast, an inverse relationship exists between body fat, visceral obesity, and antioxidant defense markers in obese individuals [11]. The hypothesis that oxidative stress is causative in the development of metabolic disorders, especially insulin-resistant state, has been supported by different studies where treatments reducing ROS production improve insulin sensitivity, hyperlipidemia, and hepatic steatosis [12–14]. Hypertrophied adipocytes have been reported as a significant source of ROS, promoting WAT dysfunction notably by altering adipokine production [12]. Furthermore, oxidative stress-associated obesity has also been shown to alter the function of many cell types or tissues (including vascular endothelial cells, myocytes, or pancreatic-*β*-cells) leading to consider oxidative stress as a contributor in obesity-related metabolic diseases.

In this review, we will expose mechanistic links between oxidative stress generated by adipose tissue in the context of obesity and its impact on metabolic complications development. We will notably focus on how WAT-associated oxidative stress might impact adipose tissue metabolism leading to WAT dysfunction. Dysregulated systemic metabolic parameters will be moreover reviewed as contributors and amplifiers of obesity-associated oxidative stress. Finally, we will discuss strategies of potential therapeutic approaches to lower obesity-associated oxidative stress and associated-metabolic complications.

## 2. White Adipose Tissue as a Significant Source of Reactive Oxygen Species Production during Obesity

Oxidative stress results from an imbalance between the production of ROS and biological systems' ability to detoxify the reactive intermediates or to repair the resulting damages, which can impact all components of the cell, including proteins, lipids, and DNA. Examples of ROS include superoxide anions (O_2_^•−^), hydrogen peroxide (H_2_O_2_), and hydroxyl radical (OH^•^). Furthermore, reactive nitrogen species (RNS) might form by combination of nitric oxide (NO^•^) with O_2_^•−^ to form peroxynitrite (ONOO^−^) and act together with ROS to damage cells, causing nitrosative stress.

### 2.1. ROS Sources in Fat Cells

**Figure 1:**
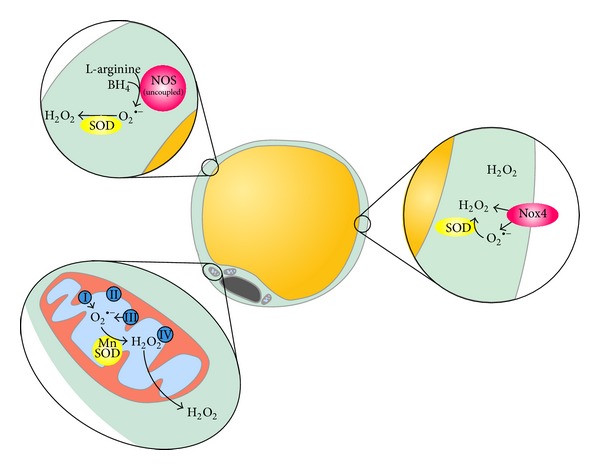
Different intracellular ROS sources participate in ROS generation by adipocytes. Mitochondria, as the core machinery for energy production through oxidative phosphorylation, is considered as a main source of O_2_^•−^, predominantly produced at complexes I and III. Superoxide anions will be further converted to H_2_O_2_ by mitochondrial manganese superoxide dismutase (MnSOD). ROS can also be produced by NADPH oxidases enzymes, in which Nox4 is the main isoform in adipocytes. This isoform presents the particularity to primarily generate H_2_O_2_, whereas other NADPH oxidases would generate O_2_^•−^ later converted to H_2_O_2_ by endogenous SOD. Nitric oxide synthase, whose eNOS and iNOS isoforms are abundantly expressed by adipocytes, might represent another source of ROS since they can be uncoupled to produce O_2_^•−^ in the absence of sufficient amounts of substrates.

Different sources of intracellular ROS detailed below might participate in ROS generation by adipocytes (Figure 1).

#### 2.1.1. ROS-Derived Mitochondria

ROS can derive from mitochondria. It is currently accepted that between 0.15% and 2% of O_2_ consumption is incompletely metabolized and results in O_2_^•−^ production, predominantly at complexes I and III [15]. Increase in nutrient uptake into adipocytes will consequently increase mitochondrial substrate load, which by increasing electron transport chain (ETC) activity will result in enhanced O_2_^•−^ by-products [16]. However, the mitochondrial origin of ROS in adipocytes is still a matter of debate. Indeed, mitochondrial metabolic flux as a basis for increased adipocyte ROS production is controversial in regard to substrate utilization in adipocytes. Free fatty acids (FFA) entering adipocytes are rapidly and predominantly converted to fatty acyl-CoA to be ultimately stored as TGs without significant mitochondrial oxidation [17]. Moreover, excess energy derived from glucose carbons undergoing glycolysis will rather enter the lipogenic pathway than the Krebs cycle, to contribute to lipid droplets fat storage [18]. In agreement with the latter, excess of glucose or palmitate does not increase mitochondrial oxidative phosphorylation or beta-oxidation in 3T3-L1 adipocytes [19].

#### 2.1.2. NADPH Oxidases

Various enzymes inside the cells can also produce ROS. Particularly, the family of NAPDH oxidases (NOX) is considered to be an important source of ROS generation [20]. NOX are membrane-bound enzyme complexes that transfer electrons from NADPH to oxygen. Generated O_2_^•−^ are further converted into H_2_O_2_, longer-lived membrane-permeable ROS, predominantly by superoxide dismutase (SOD). Seven isoforms of the catalytic subunit of NADPH oxidase have been identified in mammalian cells, known as Nox1 to Nox5, Duox1 and Duox2. NOX4, which does not require other activators and has sustained activity, is the major isoform expressed in adipocytes and is increased in fat cells exposed to excess glucose or palmitate [21]. In addition, silencing of NOX4 in 3T3-L1 adipocytes inhibits palmitate- and glucose-stimulated ROS generation underlying the importance of nonmitochondrial sources of ROS in adipocytes [19]. The molecular mechanism underlying activation of NOX by FFA has been linked to stimulated-diacylglycerol synthesis, which, connected to PKC activation, leads to the activation of NAPDH oxidase [22]. An intimate cross talk between NAPDH and mitochondria nevertheless exists since mitochondria constitute a target for ROS produced by NOX but also a significant source of ROS, which under certain conditions may stimulate NADPH oxidases [23]. It has been in fact demonstrated that mitochondria-targeted antioxidants break this vicious cycle, inhibiting ROS production by mitochondria and reducing NADPH oxidase activity [24].

#### 2.1.3. eNOS Uncoupling

Another enzyme, which may be an important contributor to ROS generation, is nitric oxide synthase (NOS) since O_2_^•−^ can react avidly with vascular NO^•^ to form ONOO^−^. Under certain pathological conditions, for instance, when the availability of NOS cofactors tetrahydrobiopterin (BH4) or substrate L-arginine is too low, the enzymatic activity of NOS can be uncoupled to produce O_2_^•−^ rather than NO [25, 26]. The transformation of eNOS from a protective enzyme to a contributor to oxidative stress has been well described in endothelial cells and observed in different models of cardiovascular diseases, including patients with cardiovascular risk factors [27]. In many cases, supplementation with BH_4_ is sufficient to correct eNOS dysfunction in animal models and patients [28, 29]. Endothelial NO synthase- (eNOS-) and inducible NO synthase- (iNOS-) dependent NO are abundant in adipocytes. iNOS expression has been shown to be increased in WAT derived from diet-induced or genetic models of obesity [30]. Similarly, both eNOS and iNOS are expressed at higher levels in WAT from obese patients compared to lean controls [31, 32]. Despite important role attributed to NO in many adipocyte metabolic functions [32], fat cell NO metabolism remains incompletely understood and contribution of eNOS uncoupling to ROS production has not been yet clearly established.

### 2.2. Enhanced ROS Production of Obese Adipose Tissues

#### 2.2.1. Elevated ROS Production by Hypertrophied Adipocytes

To determine whether fat accumulation is primarily involved in increased oxidative stress-associated obesity, Furukawa et al. have analyzed lipid peroxidation and H_2_O_2_ production in adipose tissues from obese KKAy mice [14]. They have found a specific elevation of lipid peroxidation and H_2_O_2_ production in WAT from these obese rodents, but not in liver, muscles, or aorta. Increased oxidative stress has been further described in WAT of other models of obesity such as high-fat diet [33] or* ob/ob* mice [12]. In agreement, adipocytes isolated from mice fed with high-fat diet [34] or exposed to nutrient excess* in vivo* [35] display significantly elevated ROS* in vitro*. Whereas it appears that fat accumulation parallels ROS production, as confirmed by increased ROS production during adipocyte 3T3-L1 conversion [36], contribution of the different pathways involved in this elevation of ROS is still debated. Importance of NAPDH-derived ROS are suggested by selective induction of expression of NAPDH subunits in WAT from KKAy obese mice together with the ability of NADPH inhibitors to reduce ROS production in isolated obese adipocytes [14, 34]. However, exposition of* in vitro* 3T3-L1 adipocytes to high-levels of glucose or FFA has been shown to increase mitochondrial ROS production [37, 38]. Accordingly, hyperglycemia-induced ROS production in 3T3-L1 adipocytes can significantly be reduced with pharmacological agents lowering the mitochondrial membrane potential, subsequent to overexpression of uncoupling protein-1 or SOD also suggesting a contribution of mitochondrial ROS [35]. Moreover, a number of studies have provided direct evidence of mitochondrial dysfunction associated with obesity, related to increased ROS production, supporting the development of insulin resistance [39]. Also aminooxidases, as a source of H_2_O_2_, are highly expressed in adipose tissues; they are unlikely to contribute to elevation of adipocyte production since they display reduced activities in WAT from obese subjects [40].

#### 2.2.2. Decreased Antioxidant Defenses in Obese Adipose Tissues

To prevent free radical damages, the organism has developed antioxidant defenses largely based on antioxidant enzymes able to scavenge ROS. SODs are responsible for the reduction of O_2_^•−^ to H_2_O_2_ and multiple enzymes will remove H_2_O_2_ including peroxiredoxins (PRXs), glutathione peroxidases (GPXs), and catalase (CAT). These antioxidant enzymes systems are active in fat cells isolated from rats, although their activities are lower than in liver [41]. Their activities might moreover depend on fat pad localization inasmuch as redox status differences have been reported between epididymal and inguinal fat pads from obese Zucker rats [42]. Silencing of the antioxidant enzyme glutathione-S-transferase (GSTA4) in cultured adipocytes or its invalidation in mice both results in increased ROS production and mitochondrial dysfunction [33]. These results highlight the importance of ROS scavenging processes in fat cells. Despite the presence of active antioxidant system, obese state is associated with a decrease in antioxidant defenses. For instance, a decrease in expression and activities of antioxidant enzymes such as SOD, GPX, or catalase have been reported in WAT from obese mice models [14]. Similarly, expression of the antioxidant enzyme GST-A4 in humans has been found considerably reduced in obese insulin-resistant subjects [33].

Additionally, adipose tissue represents also a preferential storage site for natural antioxidants compounds, as liposoluble vitamins (e.g., vitamins A and E) or carotenoids [43]. However, obese people present generally a relatively low total antioxidant status (TAS) characterized by lower levels of serum vitamins A, E, C, and *β*-carotene as well as glutathione [44, 45]. Although adipose tissue storage generally equilibrates with circulating levels of molecules [46, 47], fat can also act as sink concentrating vitamins in adipocyte lipid droplets therefore limiting their bioavailability [41, 48].

Altogether, sluggish behavior of the ROS scavenging system might contribute to exacerbating the existing high levels of ROS produced by hypertrophied adipocytes. In addition, the topology of the ROS production has also to be considered. Depending on detoxifying enzymes altered, they can modify ROS species and consequently downstream redox signaling.

## 3. ROS-Derived Adipocytes as Potential Instigator of Adipose Tissue Dysfunction

Numerous experimental findings favor the notion that ROS may act as second messengers triggering dedicated adaptative cellular machinery that increase the resistance of organism to stress [49]. Among ROS species, O_2_^•−^ H_2_O_2_ or NO^•^ can act as signaling molecules. For instance, in the field of aging, longevity-promoting interventions such as caloric restriction and physical activity both increase mitochondrial metabolism [50]. This activation promotes mitochondrial formation of ROS signals, which act as mild stressors, and drive an adaptative response through the induction of specific oxidative stress-sensitive pathways resulting in lifespan extension and health promotion [49, 51, 52]. In recent years, it has therefore become apparent that low levels of ROS may be required for normal cellular functioning and intracellular signaling. However, this does not negate the involvement of chronic ROS in the progression of pathologies such as diabetes. This has led to apply the theory of hormesis, concept that describes the drug action of low-dose stimulation and high-dose inhibition [50]. In addition, ROS diffusion potential is also likely to determine metabolic effects. ROS are described as short-life time species, with limited diffusion capacities especially for O_2_^•−^ and H_2_O_2_, which mainly remain in aqueous phases [53]. OH^•^ is nevertheless able to penetrate deeply in phospholipid bilayers membranes but its limited lifetime (around few nanoseconds) will considerably reduce its diffusion [54]. Therefore, depending on the degree of ROS generation, typology, and distribution, enhanced production of ROS by hypertrophied adipocytes is likely to affect metabolic pathways of cellular components of WAT as described below.

### 3.1. Oxidative Stress and Insulin Response

In adipocytes, early reports have highlighted that insulin may elicit H_2_O_2_ production in adipocytes [55]. This transient production in response to insulin involved NADPH oxidase, which produces O_2_^•−^ that spontaneously dismutates to H_2_O_2_ [56]. A number of insulin effects on adipocytes may be mimicked by H_2_O_2_ when added in physiological concentrations on fat cells [57, 58]. For instance, H_2_O_2_ enhances glucose transporters GLUT translocation and consequently glucose uptake [59] as well as lipid synthesis [55] whereas it inhibits stimulated lipolysis [60]. The rapid formation of H_2_O_2_ in insulin-treated adipocytes 3T3-L1 adipocytes causes the oxidative inhibition of protein-tyrosine phosphatases and enhances the tyrosine phosphorylation of proteins in the early insulin action cascade [61]. These data point out a novel regulatory mechanism complementing the early steps in insulin amplification signaling. Accordingly, mice invalidated for GPX1 have enhanced insulin sensitivity, effect that can be reversed by the antioxidant N-acetylcysteine [62]. This study therefore points out that subtle increase in physiological ROS production, particularly in muscle, might reflect the contribution of ROS to insulin sensitivity early in the development of insulin resistance, prior to the onset of hyperglycemia/hyperlipidemia and frank diabetes.

**Figure 2:**
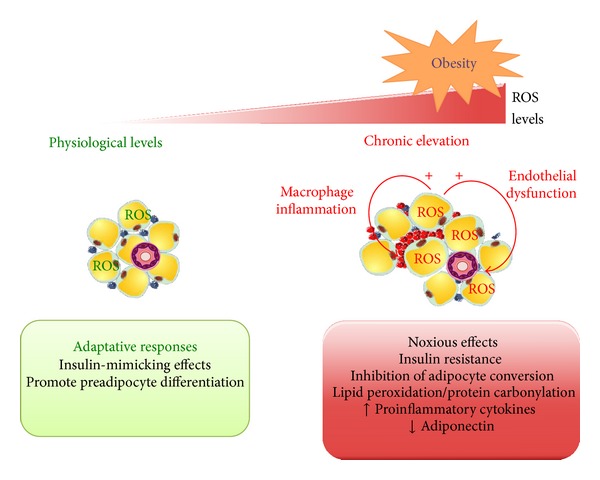
Adaptative or deleterious metabolic responses depending on ROS levels on adipose tissue metabolism. Depending on ROS intracellular levels, adipocytes will trigger different metabolic responses. Physiological levels of ROS, maintained by efficient detoxification system, are able to induce insulin-mimicking effects of H_2_O_2_ and to favor adipogenesis, which can be seen as an adaptative response in order to cope with nutrient overloading. In contrast, excessive or inappropriate redox balance will lead to considerable raise in intracellular ROS which will have detrimental effects particularly by altering insulin signaling, adipokine secretion, and adipocyte precursors conversion. Lipid peroxidation, protein carbonylation, and proinflammatory cytokines secretion are increased in obese adipocytes following exposure to high levels of ROS. Moreover, enhancement of ROS production by hypertrophied adipocytes will affect neighboring environment, namely, immune cells infiltrated in WAT or endothelial cells in close vicinity of perivascular adipose tissue. Macrophages are schematically represented as blue (M2 type) or red cells (M1) within adipocytes.

Whereas transient ROS produced by physiological stimuli such as insulin may be beneficial, sustained ROS generation impairs insulin response and might therefore initiate WAT dysfunction (Figure 2). Indeed, exposition of 3T3-L1 adipocytes to micromolar concentrations of H_2_O_2_, in response to glucose oxidase, alters the expression of GLUT transporters associated and reduced insulin-stimulated transport of glucose and lipogenesis [63]. Recent data confirm that chronic elevation of intracellular ROS levels in adipocytes subsequent to mitochondrial dysfunction results in insulin resistance through attenuation of insulin signaling [64]. Furthermore,* in vivo* evidence for the involvement of obesity-induced ROS in promoting insulin resistance has been provided using obese rodent models [12, 65]. In particular, the antioxidant MnTBAP improves insulin sensitivity in ob/ob mice that are hyperglycemic and insulin-resistant [12]. Additionally, inhibition of muscle mitochondrial ROS generation in high-fat fed rodents prevents hyperglycemia and insulin resistance [65].

Lipid aldehydes generated following lipid peroxidation of polyunsaturated acyl chains by O_2_^•−^ have also been shown to interfere with insulin signaling. Such reactive lipid aldehydes, including* trans*-4-hydroxy-2-nonenal (4-HNE), a major oxidation product containing n-6 polyunsaturated acyl groups, can covalently carbonylate proteins leading to their loss of their function. Accumulating evidences suggest important roles of these end-products in the development of insulin resistance as well as other metabolic complications associated with obesity [66]. HNE are increased in the blood and muscle tissue of obese subjects compared to normal weight subjects [67]. Of interest, expression of HNE adducts of many proteins in subcutaneous abdominal depots has been found to be higher in type 2 diabetes than in similarly obese, nondiabetic controls [66]. Muscle protein HNE adducts have been directly correlated to the severity of insulin resistance in muscle tissues [68]. They can impair skeletal muscle sensitivity by inducing oxidative damage on key components of insulin signaling [69] and inhibit glucose-induced insulin secretion [70]. Similarly, deleterious effects of 4-HNE have been reported in adipocytes. Indeed, 4-HNE impair IRS function [71], decrease adiponectin secretion [72], alter protein function following carbonylation [73], and increase lipolysis [74]. Additionally, 4-HNE might link oxidative stress and chronic inflammation in adipocytes since addition of peroxidation products to 3T3-L1 adipose cells induces cyclooxygenase-2 (COX-2) expression through p38MAPK activation [75].

Finally, exposition of adipocytes to high ROS levels suppresses adiponectin expression and secretion [14, 64], an adipokine which displays insulin-sensitizing, antiatherogenic, and anti-inflammatory properties (for review, [76]). Accordingly, human serum adiponectin levels have been inversely correlated with systemic oxidative stress [14, 77]. Regulation of adiponectin production by oxidative stress has been demonstrated* in vivo* in obese mice treated with the NADPH inhibitor apocynin, which reduces ROS production [14]. Such treatment led to improvement of insulin resistance and restoration of adiponectin production of obese rodents. This protective role of adiponectin against oxidative stress likely involved inhibition of inducible iNOS and the suppression of the expression of the gp91^phox^ subunit of NAPDH oxidase as demonstrated in experiments using myocardial infarction reperfusion model [78]. While similar effects were observed in endothelial cells [79], such a protective effect has not yet been evidenced in adipocytes. Systemic decreased levels of adiponectin will moreover contribute to maintain high levels of oxidative stress within WAT.

### 3.2. Adipose Tissue Redox Status and Adipocyte Differentiation

Fat mass expansion occurs via two processes concomitantly occurring during WAT expansion: hypertrophia (by increasing size of fat cells) and hyperplasia (by increasing numbers of fat cells linked to differentiation of adipocyte precursors). Many studies have demonstrated a close relationship between ROS and adipogenesis. Adipocyte differentiation of 3T3-L1 or rat bone-marrow derived-mesenchymal stem cells (MSC) is accompanied by a marked increase in ROS production dependent on NAPDH oxidases. In agreement, silencing of NOX4 isoform of NAPDH oxidases inhibits insulin-dependent adipocyte differentiation [80] whereas lipid storage is favored following NOX4 overexpression or exogenous application of H_2_O_2_ [81]. Conversely, scavenging of ROS production inhibits adipogenesis process [14, 82]. However, controversial data have been reported regarding ROS effects on preadipocyte recruitment and differentiation. Proadipogenic effects of ROS have been reported following physiological elevation of mitochondrial generated ROS in human MSC [83]. The latter could be related to the promotion of early mitotic clonal expansion phase of adipogenesis essential for later adipocyte conversion by ROS [36]. Conversely, antiadipogenic ROS effects have been observed when drugs increasing mitochondrial ROS production are applied to 3T3-FF42A preadipocytes, through reduction of C/EBP (CAAT/enhanced binding protein) transcription factor DNA-binding activity [84]. One hypothesis, which might reconcile such conflicting results, is based on differential effects depending on ROS intracellular levels. On one hand, “physiological” tightly regulated redox changes drive beneficial adaptative responses. On the other hand increased and uncontrolled production of free radicals might result in “noxious effects” in WAT (Figure 2). In this view, an initial burst in ROS production will switch committed preadipocytes from proliferation to differentiation. This switch can be seen as an adaptative response to nutrient overload by accelerating differentiation of adjacent preadipocytes. In contrast, excessive or inappropriate redox balance will detrimentally affect adipocyte precursors conversion therefore limiting “adipose tissue expandability” [85] and favoring adipocyte hypertrophy. Hypertrophic obesity is commonly accepted as more deleterious and associated with insulin-resistant state [86, 87]. Hypertrophied adipocytes will indeed impair adipose tissue function by inducing local inflammation, mechanical stress, and altered metabolism due to dysregulated adipokine secretion. Thus, impairment of adipocyte conversion instigated by high levels of ROS might consequently favor adipose tissue inflammation and other related metabolic dysfunctions [66].

### 3.3. Intimate Cross Talk between Oxidative Stress and Inflammation in WAT

Direct evidences from* in vitro* studies have established that adipocytes exposed to ROS upregulate expression of proinflammatory cytokines (PAI-1, IL-6) and macrophage chemoattractive molecule (MCP-1) [14]. Also, exposure of adipocytes to high levels of ROS decreases secretion of adiponectin [72]. Indeed, treatment of obese mice with antioxidants corrects adipokine dysregulation and improves diabetes, hyperlipemia, and hepatic steatosis [14]. These findings suggest that increased fat ROS in obesity is an upstream factor for adipokines dysregulation often observed during metabolic syndrome.

Additionally, many proinflammatory cytokines produced by adipocytes are able to stimulate ROS and nitrogen production by macrophages, monocytes, and endothelial cells [88]. Mitochondrial ROS production can be induced by TNF receptors signaling, following binding of TNF*α*, through TRAF2 to promote NF-*κ*B signaling [89]. Furthermore, glutathionylated lipid aldehydes, by-products of oxidative stress found to be elevated in obese WAT, are potent activators of macrophage inflammation [90]. This inflammatory state is moreover accentuated by the inhibitory effect of inflammatory cytokines on many antioxidant enzymes expression [91] or transporter selenoprotein P (SeP) known to play a role in the regulation of antioxidant enzyme activity [92]. Alternatively, immune cells infiltration might contribute to enhancing WAT-oxidative stress since ROS generated by professional phagocytes are essential components of the innate immune response against intracellular bacteria [93]. Moreover, phenotypic switch toward M1 macrophages in detriment of M2 macrophages occurring in obese WAT [94] might contribute to enhancing ROS production. Indeed, M1 macrophages secrete greater amount of proinflammatory cytokines and ROS production leading to enhance microbicidal capacity [95].

Altogether, ROS-derived hypertrophied adipocytes might initiate an inflammatory state in WAT establishing a systematic feedback-loop between inflammation and oxidative stress in obese adipose tissue concurring to WAT dysfunction.

### 3.4. WAT-Derived Oxidative Stress Could Promote Endothelial Dysfunction

Perivascular adipose tissue (PVAT) has been recently highlighted for its role in the regulation of vascular tone. PVAT can attenuate vasoconstriction based on the release of adipose tissue-derived relaxing factors [96]. In contrast, PVAT may enhance vascular contractile responses by preventing endothelium-dependent vasodilatation through inhibition of endothelial NOS (eNOS) [97]. Recent report demonstrates that PVAT promotes endothelial dysfunction in diet-induced obese C57Bl/6 mice via mechanisms linked to increased NADPH oxidase-derived oxidative stress and increased production of proinflammatory cytokines [98]. Therefore, defects in vascular tone associated with obesity might be directly related to ROS-derived PVAT on endothelium.

A major deleterious effect of ROS on endothelial cells is the decreased bioavailability of NO^•^, resulting from eNOS uncoupling [25]. NO^•^, as a potent vasodilator, is a critical component of hemodynamic regulation. Moreover, eNOS-derived NO^•^ also plays a crucial role in angiogenesis by upregulating vascular endothelial growth factors and increasing mobilization of progenitor cells from the bone marrow. Therefore, ROS-induced endothelial dysfunction will not only impair blood flow regulation but also limit capillary network formation. Such alterations will result ultimately in attenuation of microcirculatory network in metabolic active tissues and subsequent decrease in glucose utilization, particularly in WAT, muscle, and liver, key organs in the establishment of insulin-resistant state. Alteration of NO^•^-related angiogenic processes might therefore participate in inadequate vasculature observed upon fat expansion in obesity giving rise to hypoxia [99]. Another effect of oxidative stress-mediating endothelial dysfunction might rely on the deleterious effects of ROS on insulin signaling by activating stress-sensitive pathways including JNK, p38 MAPK, IKK*β* kinases, and nuclear factor-kappaB in endothelial cells (for review, [100]). Activated kinases can act on a number of potential targets in the insulin-signaling pathways, including the insulin receptor and the family of IRS proteins. For IRS-1 and IRS-2, an increase in serine phosphorylation decreases the extent of the activating tyrosine phosphorylation and is consistent with the attenuation of insulin action [101]. However, despite established roles of these different kinases in the insulin-resistant state [102–104] contribution of oxidative stress in the exacerbation of insulin resistance via IRS-1 serine phosphorylation still needs to be established. Oxidative stress, especially derived from perivascular adipose tissue, can therefore mediate endothelial dysfunction and initiate a feedback loop participating in WAT dysfunction.

## 4. Obesity-Associated Dysregulated Metabolic Parameters: Contributors and Amplifiers of Oxidative Stress

**Figure 3:**
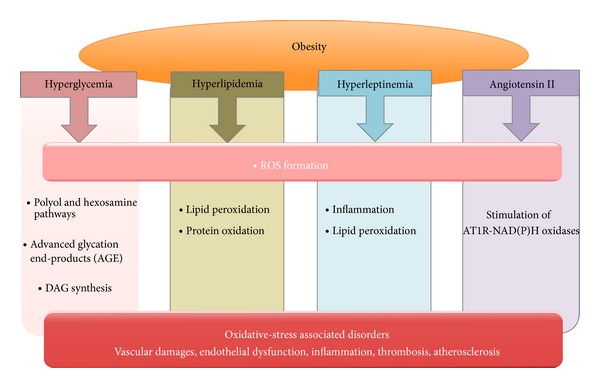
Systemic metabolic alterations associated with obesity contribute to the increase in oxidative stress. Hyperglycemia as a hallmark of type II diabetes, a metabolic complication of obesity, induces oxidative stress through activation of the polyol and hexosamine pathways, production of AGE, and increase of DAG synthesis. Excess of circulating lipids induces ROS formation pathways, which contribute to the increase in lipid oxidation and protein carbonylation. Leptin and angiotensin II, secreted at high levels by adipocytes, are inducers of ROS generation and might therefore promote inflammation and lipid peroxidation. Altogether, dysregulation of metabolic parameters occurring with fat mass expansion will contribute to inducing oxidative-stress damages notably at the vascular level.

Overproduction of proinflammatory adipokines is a trait of adipose tissue dysfunction in obesity leading to systemic inflammation in obese patients [7]. Since inflammation and oxidative stress are closely interconnected, systemic oxidative stress also appears as hallmark of the metabolic syndrome [8]. Clinical evidences for obesity-associated oxidative stress have been provided by measurement of either biomarkers or end-products of free radical-mediated oxidative processes. For instance, lipid peroxidation markers such as malondialdehyde (MDA), lipid hydroperoxides, conjugated dienes, 4-HNE, and F2-isoprostanes (8-epiPGF2*α*) are found to be increased in plasma from obese subjects in many clinical studies [9]. Of note, urinary 8-epi-PGF2*α* is strongly associated with visceral fat accumulation and BMI suggesting an important role of oxidative stress in the deleterious impact of obesity on metabolic diseases [77, 105]. Accordingly, oxidative stress has been shown to play critical roles in the development of pathologies such as diabetes, atherosclerosis, and hypertension [106, 107]. Furthermore, dysregulated metabolic parameters such as hyperglycemia, hyperlipidemia, and hyperleptinemia are important contributors and amplifiers of systemic oxidative stress (Figure 3).

### 4.1. Hyperglycemia

Diabetes mellitus is a group of metabolic diseases characterized by hyperglycemia, impaired insulin sensitivity, and developments of diabetes-induced pathologies. Hyperglycemia has been identified as the initiating cause of diabetic tissue damages seen in clinical situations including nephropathy, retinopathy, neuropathy, or vascular damages [108]. Importantly, these tissue-damaging effects of hyperglycemia are particularly noticeable in endothelial cells, since they retain expression of non-insulin-dependent GLUTs allowing intracellular glucose to rise concomitantly with extracellular glucose concentrations.

In the vascular endothelium, hyperglycemia induces overproduction of O_2_^•−^ by the mitochondrial ETC and reduces the activity of the key glycolytic enzyme glyceraldehyde-3 phosphate dehydrogenase (GAPDH) leading to the activation of three major pathways of hyperglycemic damage [109]. (1) Decreasing GAPDH activity leads to an enhancement of glucose catabolism by glycolysis and increased fluxes in the polyol and hexosamine pathways. Thereby, excess of sorbitol generated has been shown to cause cellular damage and to activate inflammatory pathways including p38 MAPK and JNK [110]. In addition, increase of N-acetyl glucosamine modifies the activity of key transcription factors leading to increased expression plasminogen activator inhibitor-1 (PAI-1) [111]. (2) Increased levels of the glycolic intermediate glyceraldehyde-3-phosphate (G3P), following GAPDH inhibition, activate intracellular production of advanced glycation end-products (AGE) precursors. Intracellular production of AGE will modify intracellular proteins or diffuse out the cells and modify matrix proteins as well as circulating proteins such albumin [16]. By binding to specific cell surface receptors (RAGE), AGE induce the production of inflammatory cytokines and growth factors, which in turn may cause vascular pathology [112]. (3) G3P elevation also leads to increased diacylglycerols (DAG) synthesis, critical activating cofactors of protein kinase-C (PKC) [113]. Increased PKC activity has been involved in vascular permeability, blood flow, and neovascularization, processes altered in diabetic patients [114]. The importance of PKC pathway in the development of diabetic complications is supported by studies showing that inhibition of PKC*β* ameliorates abnormal retina and renal hemodynamics in diabetic rats [115]. Importantly, normalization of glucose-induced mitochondrial ROS by oxidative phosphorylation uncouplers or overexpression of manganese superoxide dismutase (MnSOD) in endothelial cells prevents the activation of these three pathways of hyperglycaemic damage [116].

Alternatively, hyperglycemia also amplifies ROS production by adipocytes since high-glucose levels induce mitochondrial ROS production in 3T3-L1 adipocytes as noted previously [37, 38]. Indeed, primary adipocytes exposed to hyperglycemic conditions result in increased ROS production either* in vitro* [117] or* in vivo* [35]. Adipocytes exposed to hyperglycemia display reduced insulin sensitivity [117], partly explained by reduced insulin-stimulated glucose uptake [118] and increased inflammatory response including PAI-1 and IL-1*β* [35, 119]. Moreover, hyperglycemic clamp triggers the induction of a proinflammatory response in WAT from rats that can effectively be reduced by coinfusion of the antioxidant N-acetylcysteine (NAC) [35].

### 4.2. Hyperlipidemia

Plasma FFA concentrations are commonly elevated in obese individuals and associate with fat mass expansion [120, 121]. In healthy subjects, infusion of FFAs causes increased oxidative stress and insulin resistance, which can be reversed by infusion with antioxidants such as glutathione [122]. Elevated FFAs occurring during obesity enhance oxidative stress via increased *β*-oxidation of cytosolic long-chain acyl-CoA esters, the metabolically active form of FFAs [123]. Moreover, FFAs can also stimulate ROS production through PKC-dependent activation of NAD(P)H oxidase in aortic smooth muscle cells and endothelial cells [22]. Since ROS elicit lipid peroxidation, systemic oxidative stress will likely increase susceptibility of increased lipid pools to lipid oxidation. Accordingly, higher concentrations of 4-HNE have been found in skeletal muscles of obese persons compared to lean patients [67]. Moreover,* in vitro* oxidation of LDL and VLDL lipoproteins is significantly increased in obese, nondiabetic subjects and related to increased body weight and might be a direct consequence of lipid peroxidation-associated obesity favoring the development of atherosclerosis [124].

### 4.3. Hyperleptinemia

Leptin, WAT-derived hormone, has been shown to increase the generation of ROS in endothelial cells [125, 126]. NF-*κ*B is also activated by leptin in an oxidant-dependent manner. This effect is associated with an enhanced expression of monocyte chemoattractant protein-1 (MCP-1), which promotes atherosclerosis by favoring migration of inflammatory cells [125]. Stimulation of ROS by leptin occurs through PKC-dependent activation of NAD(P)H oxidase in vascular smooth muscle cells [127]. In addition, leptin, at concentrations similar to those found in the plasma of diabetic patients, stimulates the release of active macrophage LPL through an oxidative stress-dependent pathway suggesting a proatherogenic effect of leptin on macrophages in diabetes [128].

Another mechanism reported for leptin to induce oxidative stress is the decrease of paraoxonase 1 (PON-1) activity following its exogenous administration in Wistar rats [129]. PON-1, transported in plasma HDL, normally protects plasma lipoproteins from oxidative modification by ROS. In addition, plasma concentrations of isoprostanes as well as lipid peroxidation products are markedly increased following leptin infusion [129]. Therefore, prooxidant and PON1-reducing effects of leptin may contribute to accelerated atherogenesis in hyperleptinemic obese subjects.

### 4.4. Activation of the Renin-Angiotensin System

The renin-angiotensin system (RAS), including angiotensinogen, renin, angiotensin-converting enzyme (ACE), angiotensin II (Ang II), and its receptors, is involved in the maintenance of systemic blood pressure. In pathological state, Ang II also functions as a local biologically active mediator in the progression of cardiovascular remodeling and inflammation through Ang II type-1 receptors (AT1R). Adipocytes are a suggested source of all components of the RAS, in which production is related to obesity-associated hypertension [130]. Plasma levels of renin activity, angiotensinogen, Ang II, and aldosterone values are elevated during obesity [131]. Ang II can stimulate ROS production through AT1R-mediated NAD(P)H oxidases in different types of vascular cells [132, 133]. Moreover, AT1R blockers treatment of obese mice significantly reduces ROS originating from fat pads, attenuates the expression of NAPDH subunits in WAT, and ameliorates cytokines dysregulation [134]. Ang II-induced production of ROS is therefore an important initiator and contributor of oxidative stress-associated obesity.

## 5. Therapeutical Strategies to Reduce Oxidative Stress-Associated Obesity

Oxidative stress appears as a major contributor in the development of many metabolic complications associated obesity. Therefore, therapeutics designed to lower ROS production may have beneficial effects on health. Practically, many therapeutical strategies used currently to treat obesity-associated metabolic disorders have the potential to decrease oxidative stress, which might, at least partially, participate in their beneficial effects.

### 5.1. Caloric Restriction and Exercise

Several studies indicate that weight loss in obese patients considerably decreases oxidative stress and inflammation as evidenced by a major decrease in oxidative stress markers and decline of inflammatory cytokines [135, 136]. At the molecular level, caloric restriction activates sirtuins, NAD+-dependent deacetylases that will drive antioxidant and anti-inflammatory responses [137]. In addition, scarcity of nutrients illustrated by a low insulin/IGF-1 signaling activity enhances FOXO (forkhead transcription factor) activity by downregulating insulin-mediated FOXO phosphorylation [138] and upregulating SIRT1-mediated deacetylation [139]. Activated FOXO modulates transcription of genes involved in energy homeostasis, cell survival, inflammatory, and oxidative stress responses [140]. Finally, caloric restriction has also been shown to induce PGC-1*α* increase in mitochondria capable of efficient and balanced bioenergetics to reduce oxidative stress and ROS-associated damages [141].

Despite the paradox that exhaustive exercise might induce ROS formation, mild oxidative stress produced by regular exercise appears indeed to be able to reduce oxidative damage. Physical exercise exerts ameliorating effects on insulin resistance by increasing mitochondrial formation of ROS in skeletal muscle to induce expression of* PGC1* *α*,* PGC1* *β*, and* PPAR* *γ* as inducers of insulin sensitivity as well as SODs 1 and 2 and glutathione peroxidase 1, key enzymes of ROS defense [50, 142]. Noteworthy, regular exercise considerably improves glucose homeostasis [143] likely by restoring mitochondrial content and functional capacity of the skeletal muscle [144]. Finally, physical activity contributes* per se* to adipocyte lipid mobilization resulting in fat mass loss and repartitioning of intracellular fat, thereby improving its utilization and insulin sensitivity [145].

### 5.2. Antioxidants Diet Supplementation

Another strategy to reduce obesity-associated oxidative stress might be to supplement diet with molecules, which have proven their antioxidant capacities.

#### 5.2.1. *ω*-3-Polyunsaturated Fatty Acids

Fish oil, a major source of *ω*-3-polyunsaturated fatty acids (PUFAs), is recommended for the management of hypertriglyceridemia and to prevent from secondary cardiovascular disorders [146]. Anti-inflammatory actions of *ω*-3 PUFAs are likely mediated by the formation of their active metabolites (eicosanoids and other lipid mediators) as well as their regulation of the production of inflammatory mediators [147]. Anti-inflammatory effects of *ω*-3 PUFAs are likely linked to an inhibition of the production of eicosanoids from arachidonic acid [148].

*ω*-3 PUFAs might also exert antioxidative effects [149]. For instance, *ω*-3 PUFAS can modulate ROS production in adipocytes by impacting NADPH oxidase [19]. Moreover, supplementation of individuals with *ω*-3 PUFAs increases the expression of antioxidant enzymes and reduces the expression of prooxidant and tissue enzymes, such as cytochrome P450 enzymes and matrix metalloproteinases [150]. In adipocytes, treatment with EPA (eicosapentaenoic acid) and DHA (docosahexaenoic acid) induces heme-oxygenase 1 through the activation of Nrf-2 and prevents oxidative stress [151]. Finally, a recent study reports that the reduced macrophage infiltration is associated with increased capillaries in insulin-resistant patients following *ω*-3-acid ethyl esters supplementation. This effect contributes to damper WAT inflammation [152].

Altogether, *ω*-3 PUFA antioxidant effects associated with their anti-inflammatory properties may be of particular therapeutic value to lower obesity-associated oxidative stress.

#### 5.2.2. Vitamins

Increased oxidative stress in obesity may be exacerbated by decreased availability of antioxidants [153, 154]. This has led to the hypothesis that vitamin supplementation might benefit obese people. In agreement, daily consumption of mixed fruits and vegetable supplements, as generally preconized by dietary recommendations, significantly increases serum levels of antioxidant provitamins and vitamins (*β*-carotene, vitamins C and E) but also reduces markers of oxidative stress [155]. However, results from clinical trials regarding the beneficial effects of vitamins E, C or carotenoids in reducing the risk of cardiovascular disease are controversial since many trials failed to demonstrate any positive effects (reviewed by [156]). Conversely, adverse effects of antioxidant supplementation have even been reported such as increased risk to develop mortality [157] and cancers [158, 159]. Therefore, further studies will be needed to clearly establish the beneficial effect of vitamins supplementation on obesity-associated metabolic complications.

#### 5.2.3. Polyphenols

Polyphenols constitute the most abundant phytochemicals provided by food due to their enrichment in fruits, vegetables, and seeds-derived products. Many studies have pointed out their antioxidant and free radical scavenging properties leading to consider them as powerful antioxidants [160]. The antioxidant activity of polyphenols notably relies on their ability to inhibit ROS-generating enzymes in addition to upregulation of multiple antioxidant enzymes.

As we previously reviewed [161], polyphenols including resveratrol, quercetin, delphinidin, and red wine polyphenol extracts are beneficial in obesity-associated alterations in several animal models. We have reported that red wine polyphenol (RWP) supplementation prevents metabolic and cardiovascular alterations associated with obesity in Zucker fatty rats [162]. Particularly, RWP supplementation decreases hyperglycemia and hyperlipidemia and improves vasodilation by enhancing eNOS activity and reducing O_2_^•−^ release via decreased expression of the NADPH oxidase membrane subunit Nox-1 [162]. Resveratrol supplementation extends the lifespan of high-fat diet mice by reducing fat accumulation and improving glucose tolerance and insulin sensitivity [163]. Additionally, long-term resveratrol administration also improved metabolic disorders in obese Zucker rats and moreover produced anti-inflammatory effects in visceral adipose tissue through AMPK activation [164]. Quercetin has also been shown to improve dyslipidemia, hypertension, and hyperinsulinemia in obese Zucker rats, but only the high dose evokes the anti-inflammatory effects in visceral adipose tissue [165]. Comparing the same doses (21 mg/kg) of polyphenolic molecules (catechin, resveratrol, delphinidin, and gallic acid) in a rat model of the metabolic syndrome reveals that all molecules prevent cardiac ROS overproduction and NADPH overexpression, whereas only delphinidin and gallic acid correct insulin resistance revealing specific properties for each polyphenolic compound.

Besides their beneficial effects on lowering ROS levels, polyphenols might also influence adipose tissue mass by acting on multiple metabolic pathways (for review, [166]). Evaluation of antioxidant properties of major dietary polyphenols on 3T3-L1 adipocytes reveals that most of them inhibit proliferation of preadipocytes [167]. Curcumin and resveratrol have moreover been shown to inhibit adipocyte differentiation [168, 169]. Furthermore, polyphenol-rich grape skin extract supplementation significantly suppresses the activities of lipogenic enzymes in both adipose and liver tissues, which is concomitant with *β*-oxidation activation [170]. Polyphenols compounds also exert an anti-inflammatory effect on preadipocytes particularly by reducing inflammatory cytokines such as IL6 [167, 169]. Finally, green tea polyphenols reduce fat deposits in high fat-fed rats via erk1/2-PPAR*γ*-adiponectin pathway [171] whereas dietary black tea polyphenols prevent diet-induced obesity by inhibiting intestinal lipid absorption [172]. Mechanistically, these dietary polyphenols exert their additive and/or synergistic effects through one or more signaling and transcriptional pathways including those mediated by NF-*κ*B, AMPK, PPAR*γ*, and PGC-1*α* [166].

Despite persuasive data showing reduced body weight following treatment of obese rodents with polyphenols, only a limited number of clinical trials with supplementation of dietary polyphenols have been conducted (for review, [161]). Indeed, emerging evidence has been provided on the beneficial role of polyphenols in reducing body weight/body fat through its effects on fat and carbohydrate metabolism as well as on satiety, yet results from clinical trials are still conflicting although some results are promising. Further human long-term intervention studies are needed before recommending polyphenols in obesity management programs.

Such inconsistencies might be related to differences in chemical structure, bioavailability, and metabolism of each polyphenol tested, which may all account for different physiological effects. Moreover, despite well-established antioxidant* in vitro* activity, the biological relevance of antioxidant effects in humans is questionable in regard to their low degree of absorption and rapid metabolism within the organism. While most* in vitro* studies used polyphenol concentration range between 10 and 200 *μ*M, physiological polyphenols concentrations in humans do not exceed 10 *μ*M following a polyphenol-rich diet or after polyphenols supplementation [163]. Gut microbiota, which appeared recently to play a critical role in the development of obesity [173, 174], might moreover play a key role in bioactivity of polyphenols [175]. Taking into account qualitative changes and lack of diversities of gut microbiota associated with the obese phenotype, disturbances in polyphenol-metabolizing microbes might participate in the interindividual variability observed upon polyphenol uptake. Conversely, polyphenols might affect microbiota subpopulations by modifying redox state thereby influencing energy harvest, storage, and expenditure.

### 5.3. Thiazolidinediones (TZD)

TZD are effective oral medications for type 2 diabetes due to their insulin-sensitizing potential. The mechanism of actions of these synthetic ligands is centered on the activation of the transcription factor PPAR*γ*, a master regulator of adipocyte differentiation. Due to pleiotropic effects of PPAR*γ* activation, it is likely that the insulin-sensitizing effect would result from synergized effects of transcriptional activation of adipogenesis, glucose homeostasis, and lipid metabolism by PPAR*γ*-RXR heterodimers. PPAR*γ*  also controls the expression of many adipose-secreted factors such as adiponectin, resistin, leptin, and TNF-*α*, which influence insulin sensitivity (for review, [176]). In addition,* in vitro* and* in vivo* studies have revealed that TZD enhance mitochondrial biogenesis, notably by upregulating PGC1-*α* [177]. Importantly, rosiglitazone-treated mice display marked increased oxygen consumption and palmitate oxidation [178]. Together with increased adiponectin levels, the antioxidant properties of TZD likely participate in part in their beneficial effects.

However, safety concerns due to side effects of TZD based on increased risk of cardiovascular disorders have led to a tightly restricted access of these drugs in the United States and a recommendation for market withdrawal in Europe. Consequently, development of a new class of highly targeted and effective drugs that would preserve the strong antidiabetic efficacy of TZD but yet eliminate many of the unwanted side effects such as weight gain, fluid retention, bone loss, and heart problems is awaited. Importantly, TZD have recently been shown to induce a white-to-brown conversion through stabilization of PRDM16 protein (*P*RD-BF-1-*R*IZ1 homologous domain containing protein 16) [179]. These findings raise the possibility to transform substantial amounts of WAT to BAT, which will participate in energy dissipation rather than energy storage through UCP-1 mediated uncoupling. In this context, brown remodeling of WAT induced by SIRT1-dependent deacetylation of PPAR*γ* is promising [180]. Of particular note, activation of SIRT1 allows the cell to adapt to situations of energy stress. For instance, deacetylation of FOXO transcription factors by SIRT1 drives their actions towards the induction of oxidative stress resistance genes [139].

### 5.4. Apelin

Apelin has been recently identified as a novel peptide hormone abundantly secreted by adipocytes [181]. By interacting with G-coupled apelin receptors (APJ), apelin is implicated in various physiological functions including regulation of cardiovascular functions, fluid homeostasis, vessel formation, and cell proliferation. Of particular importance, apelin has been shown to mediate antiobesity and antidiabetics properties particularly by promoting glucose utilization and *β*-oxidation in skeletal muscles [182] and by suppressing lipolysis and adipogenesis [183, 184]. Interestingly, apelin is shown to prevent cardiomyocytes, vascular smooth muscle cells, and neurons from oxidative stress [185–187]. Antioxidant properties of apelin have been also recently reported in adipocytes [184] and excess of ROS in fat cells enhances apelin release. Apelin promotes the expression of antioxidant enzymes and suppresses the expression of prooxidant ones especially via AMPK pathway [184]. Moreover, apelin stimulates the release of adiponectin and enhances mitochondrial biogenesis. These effects contribute to counteracting oxidative-stress induced dysregulation in adipocytes.

Given the antiobesity and antioxidant properties of apelin, modulating APJ signaling appears as a promising target in the treatment of metabolic complications associated with obesity. Of particular note, ACE inhibitors or AT1R antagonists enhance apelin expression and secretion in 3T3-L1 adipocytes [188]. Therefore, the antioxidant effect reported in adipocytes following blockade of AT1R might be related to apelin action [134]. However, ACE2 enzyme has also been reported to degrade apelin-13 and apelin-36 [189]. EPA has also been described as a potent stimulator of apelin secretion in adipocytes [190]. Apelin receptors agonists are currently under development and have already proven their efficiency as vasodilator in mice [191]. Nonetheless, the use of apelin as a novel therapeutic target would need further investigations in order to better characterize properties of apelin isoforms (apelin-13, apelin-17, or apelin-36) and their bioavailability.

## 6. Concluding Remarks

Obesity is nowadays considered as a top risk factor in the development of cardiometabolic diseases and is causative of morbidity of patients suffering from metabolic syndrome. Oxidative stress has appeared in recent years as a hallmark of the obese state, intrinsically linked to chronic low-grade inflammation. High levels of ROS generated by hypertrophied adipocytes impact many metabolic signaling pathways as well as neighboring environment, such as perivascular endothelium or immune-residing WAT cells. WAT-oxidative stress therefore triggers and/or contributes to installing WAT dysfunction. Such impairment is further amplified by altered systemic metabolic parameters (hyperglycemia, hyperlipemia, hyperleptinemia, etc.) that also enhance ROS generation. Overall, systemic oxidative stress-associated obesity directly impacts insulin sensitivity of metabolic organs, promotes inflammation, and alters lipid metabolism or endothelial dysfunction. Conversely, vascular damages and inflammation participate actively in ROS generation therefore entertaining a vicious circle, which contributes to maintaining high levels of oxidative stress. Altogether, oxidative stress appears as an important contributor of metabolic diseases associated obesity such as type II diabetes or atherosclerosis.

Lowering oxidative stress to prevent such metabolic disorders therefore constitutes an interesting target. Lifestyle interventions, including dietary restriction or physical activity, have been proven to be essential in the treatment and prevention of obesity but also beneficial for ROS reduction. However, such recommendations are often difficult to implement in obese patients. An interesting aspect to explore is the potential of already available pharmacological tools (including TZD or ACE inhibitors) to reduce oxidative stress, concomitantly to their efficiency in preventing cardiometabolic risk. Whether reduction of oxidative stress might participate in their beneficial effects is however not clearly established. Future design of molecules that will further promote ROS decrease combined with their initial therapeutical properties might reveal particular interest. Another perspective is the use of antioxidants supplementation. Such a strategy can be easily undertaken since they can be extracted from natural products. Polyphenols appear as exciting molecules but further exploration would be needed to better understand their bioavailability and their molecular targets, in particular in term of genes modulated via nutragenomic approaches. Despite promising results from clinical trials, long-term studies to clearly evaluate their beneficial potential and to avoid adverse effects such as those reported for vitamins supplementation are needed [157]. Finally, one has to keep in mind that physiological levels of ROS also act as essential second messengers to maintain normal cellular functioning and intracellular signaling. Additionally, the spatiotemporal distribution of ROS and cell-antioxidant defense capacities both balance ROS intracellular levels adding to the complexity to target them. One possibility would be to target specifically ROS sources in a cell-type dependent manner. Development of mitochondria-probes molecules based on selective lipophilic cation carrier further conjugated to *α*-tocopherol (Mito VitE) or to ubiquinone (MitoQ) has already proven their efficiency to intercept mitochondrial ROS [192]. One challenge still resides in targeting specifically fat cells.

Overall, further studies are needed to clearly understand and control degree of ROS generation, typology, and distribution in metabolic tissues as well as at the whole organism level. This can lead to develop new strategies to specifically decrease ROS levels in adipocytes.

## Abbreviations

ACE: Angiotensin-converting enzyme
AGE: Advanced glycation end-products
AT1R: Ang II type-1 receptors
BH4: Tetrahydrobiopterin
BMI: Body mass index
DAG: Diacylglycerol
DHA: Docosahexaenoic acid
EPA: Eicosapentaenoic acid
eNOS: Endothelial nitric oxide synthase
ETC: Electron transport chain
FFA: Free fatty acids
FOXO: Forkhead transcription factor
GAPDH: Glyceraldehyde-3 phosphate dehydrogenase
Gpx1: Glutathione peroxidase 1
H_2_O_2_: Hydrogen peroxide
4-HNE: 4-Hydroxynonenal
iNOS: Inducible nitric oxide synthase
LDL: Low density lipoprotein
MDA: Malondialdehyde
MSC: Mesenchymal stem cells
NAC: N-Acetylcysteine
NOX: NAD(P)H oxidase
NO^•^: Nitric oxide
PKC: Protein kinase C
PUFA: Polyunsaturated fatty acids
PVAT: Perivascular adipose tissue
RAS: Renin-angiotensin system
RNS: Reactive nitrogen species
ROS: Reactive oxygen species
SOD: Superoxide dismutase
O_2_^•−^: Superoxide anions
OH^•^: Hydroxyl radical
ONOO^−^: Peroxynitrite
TGs: Triacylglycerols
VLDL: Very low density lipoprotein
WAT: White adipose tissue.
